# Supplementary material for: Microglia and Chek2 contribute to sex-specific organization of the adult zebrafish brain
Source: bioRxiv. 2025 Aug 21:2025.08.15.670359. Preprint. [Version 1] doi: 10.1101/2025.08.15.670359 (PMC12393401; doi:10.1101/2025.08.15.670359)
Supplement: Supplement 1 [file media-1.pdf]

Microglia and Chek2 contribute to sex-specific organization of the adult zebrafish brain

Paloma Bravo[1] and Florence L. Marlow[1*]

[1]Department of Cell, Developmental and Regenerative Biology. Icahn School of Medicine at Mount Sinai. New York, NY, USA

[*]Corresponding author. Email: florence.marlow@mssm.edu

Supplemental Figures and Legends

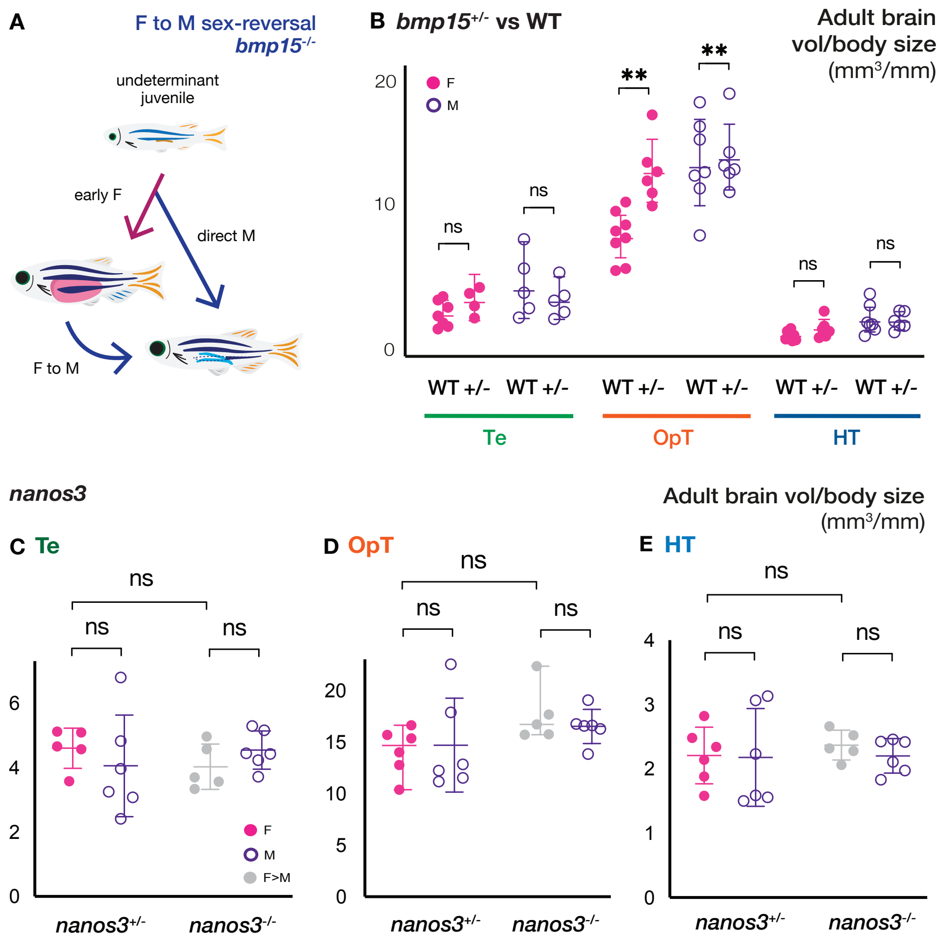

Suppl. Figure 1 Anatomical comparison of brains of sex-reversal mutants to WT

(A) Schematic depicts indeterminant juvenile stage and subsequent sex reversal associated with loss of *bmp15* . **(B)** Comparison of indicated brain region volumes between WT and *bmp15*$^{+/-}$ adult females and males. **(C-E)** Comparison of **(C)** Te, **(D)** OpT, and **(E)** HT brain volumes to body size of adult *nanos3* heterozygous and homozygous mutants. Te: telencephalon; OpT: optic tectum; HT: hypothalamus. Statistical analysis: ordinary TWO-way ANOVA with 95% CI, and Fisher LSD test for multiple comparisons. P-values: ns≥ 0.05, **<0.01.

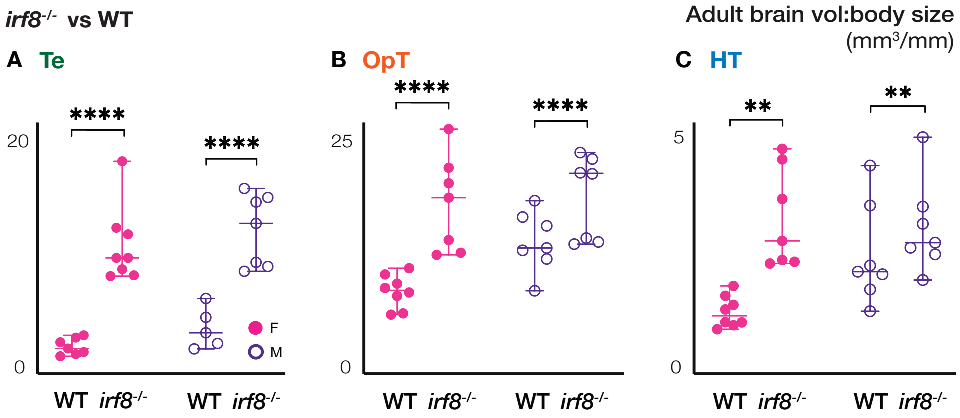

Suppl. Figure 2 Anatomical comparison of *irf8* mutant and WT brains

(A-C) Volume/body size comparison of *irf8*$^{-/-}$ and WT female and male brains **(A)** Te, **(B)** OpT, and **(C)** HT. Te: telencephalon; OpT: optic tectum; HT: hypothalamus. Statistical analysis for volume comparisons: ordinary Two-way ANOVA with 95% CI, and Fisher LSD test for multiple comparisons. P-values: **<0.01, ****<0.0001.
